# Determining Counseling Self-efficacy of Indian Students of Speech Language Pathology

**DOI:** 10.2174/17450179-v18-e2209290

**Published:** 2022-10-06

**Authors:** Nambiar Shwetha, Mohan Megha, Karuppali Sudhin

**Affiliations:** 1 Department of Audiology and Speech Language Pathology, Kasturba Medical College, Mangalore, Manipal Academy of Higher Education, Manipal, India

**Keywords:** Clinician, Counseling, Self-efficacy, Speech language pathologist, Student

## Abstract

**Purpose:**

Counseling self-efficacy is the view that counselors have of their capability to practice certain abilities that contribute to good clinical service. Speech Language Pathologists (SLPs) require to possess strategic counseling skills for effective service delivery. Although counseling is mostly considered an essential component during rehabilitation, many SLPs receive no explicit training on the same. The current study aims to explore self-efficacy measures in counseling among Indian students of speech-language pathology.

**Methods:**

The Counselor Activity Self-Efficacy Scales (CASES), which is a 6-point Likert rating scale developed by Victorino and Hinkle (2019) was adopted to assess the self-efficacy of 105 student clinicians (undergraduates and graduates) of speech-language pathology. The study comprised of two phases. Phase one included the administration of the CASES questionnaire on the target population, and Phase two included performing frequency-based analysis on Helping Skills (HS), Emotional Support Skills (ESS), and Session Management Skills (SMS) domains.

**Results:**

The majority of participants felt somewhat confident over questions in the HS and ESS domain, while a large proportion felt very confident over the questions in the SMS domain. Although the student clinicians felt somewhat confident and very confident in most of the domains, none of the participants were completely confident in any of the domains.

**Conclusion:**

Having a counseling self-efficacy tool will help estimate the level of counseling competency one may possess. The results of the study can be used to design effective counseling-based training programs for student clinicians and practicing professionals, to achieve productive therapeutic connections with patients and caregivers.

## 1 INTRODUCTION

The American Speech-Language-Hearing Association (ASHA) [1] recognizes counseling as an essential component of speech-language pathology/audiology and has included it in its scope of practice in 1997, reaffirming it in 2016. Counseling is a key clinical practice provided by Speech Language Pathologists (SLPs) and audiologists, deemed critical for effective service delivery [2]. Counseling clients and caregivers of clients with communication problems helps with painful emotions that may accompany these limitations and the development of constructive therapeutic relationships. An efficient counseling process is undoubtedly determined by the level of self-efficacy the clinician may possess towards the same. Counseling Self-Efficacy (CSE) is the view counselors have of their capability to practice certain abilities that contribute to good clinical service. The CSE can be nurtured by opportunities to learn long-term therapy practices [3]. Other researchers suggested self-efficacy as a recognized indicator of progress in any area that has a positive effect on work-related success [4]. Having high self-efficacy contributes to the growth in the effort to perform a particular task, while low self-efficacy may lead to task avoidance [5]. The measurements of self-efficacy of students increase with reading, practice, observation, and receiving feedback on their skills. Understanding self-efficacy in clinical scenarios increases preparation for graduate school, nurtures mastery of skills, and creates trust during service delivery. Having a strong self-efficacy contributes to persistence and endurance in achieving educational and career requirements [6]. Although self-efficacy measures are often confused with treatment success, researchers indicated greater levels of self-efficacy to be proportionate to learning and practice opportunities, thereby enhancing the capacity to perform skills [7].

Students and practicing clinicians of speech-language pathology require to develop counseling skills to serve clients and their families effectively. The students, during their graduate training years, are provided with the basic knowledge about the services and practices necessary to manage their clients. This training includes collecting information, supplying data, and interviewing clients [8]. The SLP further initiates counseling interviews to influence attitudes to enable clients/caregivers to recognize the communication disability. Since counseling sessions involve the emotional regulation of clients, student clinicians must thereby cultivate a client-centered rather than a clinician-directed focus that encompasses learning to accept calmness during the clinical interaction.

Johnson and Van Riper acknowledged the influence and importance of therapy outcomes resulting from the quality and kind of interaction relationship between SLPs and their patient(s) as early as 1950. This brought attention to the need for more widespread integration of counseling into speech-language pathology practice, as well as the need for enhanced practitioner comfort and expertise in doing so. Researchers investigated the clinical self-efficacy skills of students of speech-language pathology during their academic year, and found a relationship between clinical performance and self-efficacy, and the effects of peer modeling on clinical skill development [9]. The results indicated moderate clinical self-efficacy skills among student clinicians, with a statistically significant relationship with the performance ratings of their clinical supervisors, and a strong link between clinical performance and clinical self-efficacy. Additionally, the study found clinical self-efficacy in speech-language pathology to vary with clinical experience, with a substantial positive relationship evident between clinical self-efficacy beliefs and the number of direct contact hours. This was apparent with a significant difference observed between the speech-language pathology clinical self-efficacy beliefs of first and second-year graduate-level students. On similar lines, researchers found the capability of students of speech pathology to deal with individuals who stutter to have improved distinctly portrayed through clinical self-efficacy measures [10]. However, the relationship between the clinical self-efficacy of students and their clinical skill assessment by their clinical supervisors was found to be lower. Similar relationships between counseling students self-efficacy, clinical experience, and clinical competency were reported in other research as well [11].

Expertise and experience are dominant sources of self-efficacy information, implying second-year students gain confidence in completing clinical tasks through practical experience and observation, compared to first-year graduate students who received even and measured experiences in university clinics [5]. These graduate students eventually participate in university-based training programs where they excel in managing difficult patients in an assortment of clinical settings by sheer peer observation. Researchers reported an improvement in the clinical self-efficacy of students of speech-language pathology with enactive mastery. These enactive interactions affected self-efficacy through cognitive processing of knowledge about the accomplishments and shortcomings of the person. Clinical supervisors identified an important optimistic relationship between speech-language pathology clinical self-efficacy and the clinical results and found a clear positive correlation between self-efficacy and people's success on specific tasks.

Studies have documented self-efficacy during self-regulated learning to have an optimistic association with seeking help and problem-solving behaviors. Interestingly, the origins of self-efficacy change during the learning process [12]. Participants initiated more help-seeking behaviors on certain tasks, with high self-efficacy levels attained over tasks with a successful history. However, participants were able to use more problem-solving behaviors as self-efficacy improved with previous performances. This suggested people with higher levels of self-efficacy to initially seek support from others but with skill mastery, excelled in problem-solving skills. Brain responses from task performance and cognitive processes tests indicated a positive relationship between self-efficacy with better precision skills, task conclusion, and faster reaction times, when planning for or reacting to a specific stimulus and the ability to provide more concentration and attention during task implementation [13].

Continued education to elucidate basic counseling constructs and in achieving affirmative outcomes becomes critical to the expansion of the clinician’s role. Researchers indicated the existence of few courses in counseling in graduate-level programs, aiding a small proportion of graduates to receive counseling training [14]. Increasing the appropriate integration of counseling into speech-language pathology practice undoubtedly necessitates continued research and experience to improve counseling applications, skills, knowledge, approaches, and techniques [15]. Although counseling in speech-language pathology is similar to counseling in any other professional field, SLPs receive less training during their graduate program. Eighty-two percent of graduate students of speech-language pathology were reported to require counseling preparation programs, and more clinical experience in counseling practice during their training programs [16]. The ASHA does urge SLPs to develop brilliant counseling skills and assistance for the patient’s psychological and emotional concerns and specified that although exceptional counseling skills are recommended, they are not always evident in practitioners [17]. This has resulted in high demand for SLPs to develop good emotional, social, and psychological support systems for their clients [18]. On these lines, there exist no such standard counseling-based programs which are integrated within the curriculum of speech-language pathology in India, nor are there any counseling-based programs offered for clinicians handling individuals with communication disabilities. With the delivery of counseling services not being the primary objective of clinical practice, clinical supervisors tend to expect student clinicians to have exceptional counseling skills throughout their training years, irrespective of the nature of the disability they deal with. Considering the future ramifications of not having trained student clinicians on delivering good counseling services for their clients, it becomes highly imperative to profile the counseling self-efficacy in these individuals. Having studied their clinical limits, gives a clear understanding of the training requirements to overcome this issue. Nevertheless, given the benefit of applying such fundamental counseling principles in speech-language pathology, and evaluating the presumed counseling self-efficacy in SLPs, does help predict therapy effectiveness, ensuring the best clinical service for patients and caregivers. The Counselor Activity Self-Efficacy Scales (CASES) does evaluate the self-efficacy of counseling students [19]. This 6-point Likert rating scale helps determine the student's self-assurance in their ability to deliver different clinical services during the academic program. Therefore, the objective of the current study was to explore the self-efficacy measures in counseling among final year undergraduate and final year graduate students of speech-language pathology using the CASES questionnaire.

## 2 MATERIALS AND METHODS

The current study aimed to study the self-efficacy measures in counseling among the students of speech-language pathology. The study followed a cross-sectional study design with a convenience sampling method. The study was done in accordance with the Helsinki Declaration of 1975, as revised in 2008. The protocol was approved by the Institutional Ethical Committee of KMC, Mangalore.

### 2.1 Participants

The sample size for the current study was calculated by referring to a study [20], and using the formula n=[Z_1-α/2_+Z_1-β/2_/c]^2^, where *Z*_1-*a*/2_=1.96 (with 95% Confidence level) maintaining 80% power, c=0.3196, and r=0.435. Out of 111 students who were contacted for the study, a total of 105 responded and were included in the present study. The participants comprised of final-year undergraduates and final-year graduate students of speech-language pathology. The participants were recruited from various institutes across South India. Table **1** shows the demographic details of the participants of the current study.

Before the conduction of the research, the participants were explained the purpose of the study. The inclusion criteria included students currently undergoing their internship and students in the final year of their post-graduation program in speech-language pathology, pursuing the same from any institution (private or government). The exclusion criteria included graduate students with any prior work experience, students who have received any form of formal training in counseling, and students with any other academic degree.

### 2.2 Cases

The CASES questionnaire [19] was originally developed to assess the self-efficacy of counseling students. The questionnaire was designed to explore the conceptualization of counseling skills using three domains: (a) Helping Skills, (b) Session Management, and (c) Counseling Challenges. Victorino and Hinkle modified the original version of the CASES questionnaire [20] to explore the self-efficacy of students of speech-language pathology in counseling, and by retaining the domains of ‘helping’ and ‘session management, and replacing the domain of ‘counseling challenges’ with ‘emotional support’. Permission was obtained from the authors and the Publishers of the American Journal of Speech-Language Pathology to use the questionnaire for the current study.

The questionnaire planned for the current study included three domains of assessment. The first domain incorporated the Helping Skills (HS) domain (13 items) which included Helping Skills Exploration [HS(E)] (5 items), Helping Skills Insight [HS(I)] (4 items), and Helping Skills Action [HS(A]) (4 items). The HS(E) subdomain comprised of questions on basic communication competencies such as attending, listening, asking open questions, restatements, and reflecting client’s feelings. The HS(I) subdomain comprised of questions on more complex counseling behaviors about challenging client inconsistencies, intentional silence, interpreting what the client has overtly stated, and offering immediacy statements. Lastly, the HS(A) subdomain comprised of questions on more basic communication competencies such as providing information, direct guidance, providing the best response, and helping the client/caregiver to explore his/her thoughts. The second domain included Emotional Support Skills (ESS) (10 items) comprising of questions specific to emotions experienced by clients/families with communication disorders, such as helping the clients/caregivers in providing an appropriate response to various emotions such as grief, anger, guilt, denial, resistance and also providing empathic responses to the concerns of the clients/caregivers. The final domain included Session Management Skills (SMS) (10 items) comprising of questions on session management such as providing structure to sessions, asking questions to evaluate treatment progress, maintaining professional boundaries, knowing how to address sensitive topics, asking open questions, and providing clients/caregivers with appropriate referrals when necessary. A domain to extract the demographics (educational history, clinical experience, and any history of clinical courses in counseling) was also included in the questionnaire. The response system included a 6-point self-rating Likert scale (0-5) ranging from ‘not familiar with the concept’ to ‘completely confident’.

### 2.3 Procedure

The study was conducted in two phases: Phase I included the administration of the CASES questionnaire to the two groups (undergraduates and graduates), and Phase II included data and statistical analysis of the retrieved data. In Phase I, the modified CASES questionnaire [20] was adapted into an online survey (Google Form). Following the permission obtained by the institutional management, the participants were contacted and provided with an email link to the survey. The survey link included the objective of the study and the informed consent statement. Participants who consented to take part in the study were asked to fill out the CASES questionnaire. Multiple reminders were sent for the survey completion. The first reminder was sent seven days after receiving the online survey link, followed by the second one sent three days later, and the final one a day later.

In Phase II, following the administration of the CASES questionnaire, the data of the two groups of participants were analyzed. The dependent variables were the domains – HS [(HS(E), HS(I), and HS(A)], SMS, and ESS. Descriptive statistics were used to determine the n (%) of the participants who rated their self-efficacy under each of the domains. In addition to this, the strength of association between the perceptions of undergraduate and graduate students across each of the items under HS, SMS, and ESS counseling domains was measured using the Chi-square test.

## 3 RESULTS

The study aimed to explore self-efficacy measures in counseling among students of speech-language pathology. The results are depicted based on each domain of interest.

### 3.1 Helping Skills Domain

A small proportion of participants felt *completely confident* in the HS(E) domain, with a majority of them feeling *somewhat confident* and *very confident* over the same. However, a high level of confidence was observed towards questions addressing exploratory-based statements such as attending, asking open questions, restating the statements, and reflecting clients’ feelings. On a similar note, a large proportion of the participants felt *somewhat confident* and *very confident* over the HS(I) domain, towards questions related to challenges, providing intentional silence, and so on, while a certain number of participants indicated a low level of confidence in complex counseling behaviors, such as challenging client inconsistencies and offering immediacy statement, addressing the challenges faced by the participants and so on. As for the HS(A) domain, a majority of the participants felt *somewhat confident* and *very confident* for questions on information-giving and providing direct guidance to the clients/caregivers. Table **2** illustrates the responses of the participants toward the HS domain.

### 3.2 Emotional Support Skills Domain

Although a small proportion of the participants felt *completely confident* in the ESS domain, a majority of them felt *somewhat confident* and *very confident* over the same. A high level of confidence was observed for questions addressing emotions such as expressing feelings of grief, anger, guilt, denial, and resistance. A low level of confidence was attributed to questions involving more complex behaviors, such as understanding the thoughts, feelings, and actions, deciding what actions to take regarding the problems, and knowing what to say after the patient expresses their feelings. The following table Table **3** illustrates the responses of the participants toward the ESS domain.

### 3.3 Session Management Skills Domain

The majority of the participants felt *somewhat confident* and *very confident* over the SMS domain. A high level of confidence was observed for questions regarding the provision of structure to sessions, evaluation of treatment progress, along with other content or information-oriented items such as providing clients/caregivers with appropriate referrals when necessary. On a similar note, a large proportion of participants felt *completely confident* in this domain. Certain participants did indicate a low level of confidence towards the involvement of more complex behaviors, such as engaging in discussions related to feelings expressed, maintaining appropriate professional boundaries, knowing how to address sensitive topics, and so on. Table **4** illustrates the responses of the participants regarding the SMS domain.

The results of the Chi-square test revealed a poor level of significance (p<0.05) between undergraduate and graduate students across all the items under HS, SMS, and ESS domains.

## 4 DISCUSSION

### 4.1 HS Domain

The varying levels of confidence reported by the clinicians in the HS(E), HS(I), and HS(A) subdomains depended on the nature of the questions asked. Considering the HS(E) subdomain, the ‘attending’ (Q1) and ‘listening’ (Q2) related questions targeted the perception of clinicians getting involved in counseling without any active verbal participation, compared to Q3 (restatements), Q4 (open questions), and Q5 (reflections of feelings) which was otherwise. The student clinicians (77%) reported higher levels of confidence in active listening, followed by 51% in actively attending to the client. Although explicit verbal questions such as using open questions, providing restatements, and engaging in reflections of feelings reported higher levels of confidence by the clinicians (47-58%), listening remained the skill that clinicians felt most confident in. A smaller proportion of clinicians reported possessing lower levels of confidence in reflections of feelings (19%), open questions (10%), restatements (9%), attending (7%), and listening (3%) related aspects. Research claims clinicians willing to compassionately listen and attend to the client’s problems did indeed provide compassionate care to adults, seeking to improve their lives without burdening themselves or the clinicians [18]. Researchers indicated that student clinicians with greater reflective listening skills received higher positive evaluations of their counseling skills from their superiors, compared to clinicians with lesser reflective listening skills [21]. Having such active listening skills helps convey empathy rather than sympathy [22]. Hence, the author recommends that SLPs avoid statements such as “I know how you must feel”, and instead use more supportive statements like “Let’s talk about those feelings”, to build a trusting relationship between the SLP and the family.

In the HS(I) subdomain, like Q2, the Q6 (intentional silence) too did not involve any active verbal participation by the clinician, thereby observing the highest level of confidence (44%) in the same, followed by Q8 (interpretations), Q7 (challenges), and Q9 (immediacy) respectively. A larger proportion of clinicians reported possessing lower levels of confidence in disclosing immediate feelings (35%), managing challenges (27%), providing interpretations (24%), and engaging in intentional silence (18%). Considering the low confidence levels reported by clinicians in making statements that may go beyond what the client has overtly stated, clients find it challenging to appreciate his or her behaviors, thoughts, or feelings from the perspective of an SLP. In line with this, it was found that patients were experiencing times of severe stress unlikely to utilize effective coping strategies to move through their emotions, with the SLP playing an important role in recognizing and understanding the family’s grief, thereby facilitating positive strategies to deal with the same [23]. With clinicians reporting higher moderate levels of confidence in disclosing immediate feelings to the client, evidence concluded that such disclosure of causal information provides opportunities to reevaluate the therapeutic relationship, thereby changing the patient’s negative feelings [24]. The author further recommends health care workers make disclosures not only in the case of unavoidable causes but also in avoidable ones.

The HS(A) subdomain indicated a larger proportion of clinicians to report higher levels of confidence in Q10 (information giving), Q11 (direct guidance), and Q13 (helping explore thoughts/feelings). Comparatively a lower proportion of clinicians (39%) reported possessing high levels of confidence in Q12 (providing the best response). The knowledge and experience clinicians possess directly or indirectly influence the counseling strategies indicated in Q10-13. Additionally, having direct exposure to clients influences self-confidence/self-efficacy more than any simulated context [25]. However, providing simulated contexts to students improved their confidence in applying the nursing process, organizing nursing care, and performing technical skills more than having a clinical experience, which consecutively increased the students’ level of confidence in the ability to use those skills with real people. The requirement of general counseling considerations, along with specific suggestions, may help practitioners expand their confidence levels during clinical practice [26].

The overall average proportion of clinicians received higher levels of confidence in HS(E) (30%), followed by HS(A) (28%) and HS(I) (25%). The higher level of confidence in the HS(E) domain can be attributed to the nature of the questions in this domain which targeted basic communication competencies, compared to the questions of the HS(A) and HS(I) domains which focused on complex counseling behaviors.

### 4.2 ESS Domain

The ESS, unlike the HS domain, involved the emotional aspects of counseling, with a larger proportion of clinicians (36-49%) reporting higher levels of confidence across all items, with the highest observed in Q22 (providing empathic responses to the concerns) (49%), followed by Q14 (helping understand thoughts/feelings) and Q15 (helping decide what actions to take) (47%), and Q16 (response to clients expressing grief) (45%). A larger proportion of clinicians (27%) reported lower levels of confidence in Q21 (counseling a client/family member regarding their locus of control and how it relates to their feelings/attitudes about their communication disorder), followed by Q20 (providing an appropriate response to clients or family members expressing feelings of resistance regarding their communication disorder). The ability to engage empathically with clients develops naturally as a result of accurately identifying and communicating client feelings, and also considering that empathy is a multifaceted process beginning with an effective response (*e.g*., a gut feeling of anxiety) and progressing towards a cognitive response (*e.g*., reflecting feeling) [3]. As a result, being able to empathize does necessitate the ability to suspend judgment and bias to walk in the shoes of another. Student clinicians provided crucial information in assessing strengths and weaknesses across domains that might influence decision-making for communication intervention, as well as advising changes for other service providers [27]. Therapists found it challenging to uncover parts of counseling involving exploring and expressing the client’s feelings, as well as generally opening up the entire area of inner experience for exploration [28]. The latter phase involves becoming aware of the implications of the loss, and how the person has coped personally with the same.

### 4.3 SMS Domain

The SMS domain involved questions about session management, with a larger proportion of clinicians (52-58%) reporting higher levels of confidence across the majority of the items, with the highest observed in Q25 (evaluating clients' progress) (59%), followed by Q27 (maintaining appropriate professional boundaries with the client) (58%), Q31 (answering questions regarding diagnosis and treatment clearly) and Q33 (obtaining information regarding the impact of the communication impairment on the family system) (53% each), Q26 (providing clients with appropriate referrals) and Q32 (obtaining information regarding the impact of the communication impairment on the client’s life and relationships) (52% each). A larger proportion of clinicians (31%) reported the lowest level of confidence in Q29 (engaging the client in a discussion related to his or her culture and its impact on the feelings, or actions related to the communication disorder), followed by Q28 (addressing sensitive topics) (30%) and Q30 (engaging families as co-diagnosticians in the diagnostic process) (21%). The SLPs actively lead the journey by examining communication behaviors, setting goals with clients, teaching new skills, and documenting progress, as well as clinicians being completely present and listening empathically without passing a judgment [29]. It becomes highly essential to maintain good interactions and prevent communication breakdowns among clients [30]. This can be achieved by the involvement of all partners (including the health care professional) in the communication situation, addressing the communication strategies (anticipation, repair, and maintenance strategies) and emotional behaviors. Counseling clients and family members of clients with communication problems aid in not just coping with the tough emotions that come with these impairments, but also in developing positive therapeutic relationships [20]. The therapeutic relationship can be defined as one in which the client is respected as an individual capable of thinking, feeling, and making informed decisions, while the clinician serves as both a teacher and a supporter [31]. Clinical professions of nursing and occupational therapy have reported clinical traits such as interpersonal skills, attentiveness, care, and warmth, to contribute to increased client evaluations for successful therapy [28].

### 4.4 General Discussion

Multiple variables hinder clinicians from obtaining the highest levels of confidence in HS, SMS, and ESS domains. The clinical exposure of the participants in the current study may have contributed immensely to their self-efficacy rating. The coursework on speech-language pathology in India is regulated by the Rehabilitation Council of India, with the bachelor’s and master’s level programs offered in different institutes/universities across India. Depending upon the nature of the organization, the undergraduate and graduate students begin acquiring clinical exposure either from hospitals, rehabilitation centers, clinics, schools, or other clinical setups. The clinical exposure obtained from either of these systems or a combination of them eventually influences one’s clinical preparedness in counseling. An association between the level of clinical experience of graduate students and their clinical self-efficacy has been reported [9]. They reported the clinical self-efficacy of the SLP students to have improved after the commencement of their first academic year of clinical practice. Self-efficacy is indicated to be an important predictor of satisfaction with clinical experience among students of speech and language pathology [32]. Researchers suggested student clinicians focus their attention on their clinical work, which increasesthe pressure on the amount of preparation required, thereby helping fulfill high expectations people may have on them [33]. The current study included undergraduate clinicians who were in their final (fourth academic) year, and graduate clinicians in their final (second academic) year, which does brand them as ‘experienced clinicians’. However, as observed, their overall self-efficacy confidence ratings in helping exploration, emotional support, and session management did not reach high levels as anticipated. Experience-guided counseling efficacy has been reported in first-year therapists who exhibited lesser counseling competencies than fourth-year therapists [31]. As a result, students are generally provided with a clinical practicum to offer information and skills through enactive experiences. Enactive experiences are of utmost influential contributors to the development of self-efficacy [34], as they comprise the most authentic evidence of competencies [35]. Studies have shown students to feel emotionally unprepared and anxious during clinical practice, thereby hindering their ideas and thoughts about the implementation of appropriate intervention and modification of materials [36]. Student clinicians who have gained clinical experience, appeared to exhibit a reduction in fear and avoidance while working with patients [10]. Anxiety and stress are recognized as contributing factors in the clinical practice of undergraduate SLP students impacting their counseling services [26]. Stress impacts effectiveness, productivity, attitudes, professional behavior, and job satisfaction. Researchers studied 127 speech-language pathology students (second, third and fourth years of a university degree program) and identified several factors contributing to the anxiety levels in each year of the program [37]. Some of the significant contributors were applying theory to practice, having high expectations of themselves, the amount of clinical experience and preparation required, and fulfilling university and clinical demands simultaneously. However, these anxiety levels progressively decreased as the students reached their final year of education. Another interesting, yet unsurprising observation in the current study was the under-representation of males (6.67%) over female (93.33%) participants. The lack of male SLPs have been a persistent concern in the field of speech-language pathology [38]. Although there exists no established study on the gender inequality in Indian SLPs, the dominance of female students opting to pursue their profession in speech language pathology is quite apparent in institutions offering such programs throughout India. Such issues restrict the ability to best serve a diverse clinical population creating major health disparities [39]. Though controlling for gender was not within the scope of the current study, it would be interesting to explore the counselling efficacy between male and female student clinicians, which could possibly be considered as a future direction.

One another primary and universal element leading to the poor preparedness of student clinicians in providing effective counseling services can be attributed to the ongoing COVID-19 pandemic. Although graduate SLPs are deemed to have an upper edge over undergraduate SLPs in counseling skills, the same was not true in the current study. A poor level of significance (p<0.05) was evidently using the Chi-square test, indicating both student groups to have equal competency in all three counseling domains. The final year undergraduates who participated in the current study were academically enrolled in their bachelors’ program in late 2018. Considering the first year (first and second semesters) of their undergraduate program to focus more on theory and clinical observation, these students began their second year (third and fourth semesters) late in 2019. With only a couple of months of clinical exposure, the Indian government, in the year of March 2020 declared a nationwide lockdown, which included the closure of all educational institutions, including clinical setups. This sudden halt in the clinical exposure of students resulted in severe educational challenges students encountered [40]. Although eventually there were systematic educational and clinical services openings, social distancing and wearing masks were made mandatory. With multiple regulations in place, these student clinicians started receiving limited clientele, who were irregular due to the health concerns they had while traveling to obtain such services [41]. Referrals to speech-language therapy providers in the UK during the acute COVID-19 period were reported to be significantly lower than in the same period in 2019 [11]. Subsequently, with the second lockdown declared by India in 2021, these student clinicians entered their internships by mid-year. On the other hand, the graduate students completed their 2-year (2019-2021) Master’s program in speech-language pathology amidst the pandemic. With poor clinical exposure, these students manifested counseling skills at par with undergraduate clinicians, eventually managing patients without adequate counseling skills.

Although the present study explored the self-efficacy measures in undergraduate and graduate students of speech-language pathology, a comparison between the two student groups was out of the scope of this study. Future research may consider to address this issue, as well as plan for a larger sample size, considering the number of speech and hearing institutes present across India. The results of the study can be used to design effective counselling-based training programmes for student clinicians and practicing professionals, to achieve productive therapeutic connections with patients and caregivers. According to the preferred practice patterns for the profession of speech-language pathology [1] counselling must involve providing appropriate information and direction to patients, caregivers, families, and other relevant individuals about the nature of the disability (s), the course of management, methods to enhance results, coping with disability(s), and prognosis. On the contrary, there are no guidelines recommended by the Indian speech and hearing association on providing counselling services by SLPs.

### CONCLUSION

Although counseling is viewed by most professionals as an essential component of rehabilitation, most SLPs receive no explicit training in the same. Having a counseling self-efficacy tool will help in estimating the level of counseling competency one may possess. This will help predict the effectiveness of the delivered therapy sessions and in estimating the overall communication abilities with patients and caregivers, ensuring the best care that can be provided to them. The results of the study can be used to design effective counseling-based training programs for student clinicians and practicing professionals, to achieve productive therapeutic connections with patients and caregivers.

## Figures and Tables

**Table 1:** The demographic details of the participants of the current study.

**Educational Level**	**Gender**		**Total** **n (%)**	**Mean Age** **(in years)**
	**Males** **n (%)**	**Females** **n (%)**		
UG	4 (7.69)	48 (92.30)	52	21.71(±1.25)
Graduates [M.Sc (SLP)]	2 (3.77)	43 (81.13)	45	23.31(1.12)
Graduates (MASLP)	1 (1.88)	7 (13.20)	8	24.5(1.51)
Total	7	98	105	22.60(1.53)

Note: UG – Final year undergraduates; M.Sc (SLP) – Masters in Science (Speech-Language Pathology); MASLP – Masters in Audiology and Speech-Language Pathology.

**Table 2:** The responses of the participants for the HS [HS(E), HS(I), and HS(A)] domain.

**Item No.**	**I’m Not Familiar with that Concept** **n (%)**	**Not at all Confident** **n (%)**	**A Little Confident** **n (%)**	**Somewhat Confident** **n (%)**	**Very Confident** **n (%)**	**Completely Confident** **n (%)**
Helping Skills Exploration [HS(E)]						
Q1	1 (0.95)	0 (0)	6 (5.71)	44 (41.90)	33 (31.42)	21 (20)
Q2	1 (0.95)	0 (0)	2 (1.90)	13 (12.38)	47 (44.76)	34 (32.38)
Q3	0 (0)	1 (0.95)	8 (7.61)	39 (37.14)	39 (37.14)	18 (17.14)
Q4	3 (2.85)	1 (0.95)	6 (5.71)	39 (37.14)	33 (31.42)	23 (21.90)
Q5	3 (2.85)	1 (0.95)	16 (15.2)	36 (34.29)	33 (31.42)	16 (15.24)
Helping Skills Insight [HS(I)]						
Q6	4 (3.80)	1 (0.95)	14 (13.33)	40 (38.09)	32 (30.47)	14 (13.33)
Q7	3 (2.85)	4 (3.80)	21 (20)	41 (39.04)	21 (20)	15 (14.28)
Q8	2 (1.90)	3 (2.85)	20 (19.04)	36 (34.28)	33 (31.42)	11 (10.47)
Q9	4 (3.80)	7 (6.66)	26 (24.76)	37 (35.23)	22 (20.95)	11 (10.47)
Helping Skills Action [HS(A)]						
Q10	2 (1.90)	5 (4.76)	10 (9.52)	28 (26.66)	43 (40.95)	17 (16.19)
Q11	3 (2.85)	4 (3.80)	9 (8.57)	31 (29.52)	44 (41.90)	14 (13.33)
Q12	2 (1.90)	1 (0.95)	13 (12.38)	48 (45.71)	33 (31.42)	8 (7.61)
Q13	3 (2.85)	3 (2.85)	7 (6.66)	39 (37.14)	41 (39.04)	12 (11.42)

**Table 3:** The responses of the participants for the ESS domain.

**Item No.**	**I’m Not Familiar with that Concept** **n (%)**	**Not at all Confident** **n (%)**	**A Little Confident** **n (%)**	**Somewhat Confident** **n (%)**	**Very Confident** **n (%)**	**Completely Confident** **n (%)**
Emotional Support Skills (ESS)						
Q14	2 (1.90)	1 (0.95)	15 (14.28)	38 (36.19)	36 (34.28)	13 (12.38)
Q15	2 (1.90)	2 (1.90)	13 (12.38)	39 (37.14)	38 (36.19)	11 (10.47)
Q16	2 (1.90)	4 (3.80)	9 (8.57)	43 (40.95)	38 (36.19)	9 (8.57)
Q17	2 (1.90)	6 (5.71)	15 (14.28)	39 (37.14)	33 (31.42)	10 (9.52)
Q18	3 (2.85)	3 (2.85)	19 (18.09)	39 (37.14)	31 (29.52)	10 (9.52)
Q19	2 (1.90)	5 (4.76)	18 (17.14)	39 (37.14)	33 (31.42)	8 (7.61)
Q20	2 (1.90)	6 (5.71)	19 (18.09)	37 (35.23)	31 (29.52)	10 (9.52)
Q21	3 (2.85)	4 (3.80)	21 (20)	32 (30.47)	34 (32.38)	11 (10.47)
Q22	2 (1.90)	3 (3.80)	13 (12.38)	36 (34.28)	36 (34.28)	15 (14.28)
Q23	2 (1.90)	2 (1.90)	19 (18.09)	44 (41.90)	27 (25.71)	11 (10.47)

**Table 4:** The responses of the participants for the SMS domain.

**Item No.**	**I’m Not Familiar with that Concept** **n (%)**	**Not at all Confident** **n (%)**	**A Little Confident** **n (%)**	**Somewhat Confident** **n (%)**	**Very Confident** **n (%)**	**Completely Confident** **n (%)**
Session Management Skills (SMS)						
Q24	2 (1.90)	2 (1.90)	15 (14.28)	33 (31.42)	36 (34.28)	17 (16.19)
Q25	2 (1.90)	3 (3.80)	10 (9.52)	28 (26.66)	38 (36.19)	24 (22.85)
Q26	2 (1.90)	2 (1.90)	5 (4.76)	37 (35.23)	31 (29.52)	24 (22.85)
Q27	2 (1.90)	3 (3.80)	6 (5.71)	33 (31.42)	36 (34.28)	25 (23.80)
Q28	2 (1.90)	7 (6.66)	22 (20.95)	31 (29.52)	31 (29.52)	12 (11.42)
Q29	3 (2.85)	5 (4.76)	25 (23.80)	34 (32.38)	26 (24.76)	11 (10.47)
Q30	3 (2.85)	4 (3.80)	15 (14.28)	40 (38.09)	30 (28.57)	13 (12.38)
Q31	2 (1.90)	2 (1.90)	15 (14.28)	30 (28.57)	39 (37.14)	17 (16.19)
Q32	2 (1.90)	2 (1.90)	15 (14.28)	31 (29.52)	40 (38.09)	15 (14.28)
Q33	2 (1.90)	2 (1.90)	16 (15.23)	29 (27.61)	43 (40.95)	13 (12.38)

## Data Availability

The authors confirm that the data supporting the findings of this study are available within the manuscript.

## LIST OF ABBREVIATIONS

SLPs: Speech Language Pathologists
CASES: Counselor Activity Self-Efficacy Scales
HS: Helping Skills
ESS: Emotional Support Skills
SMS: Session Management Skills
ASHA: American Speech-Language-Hearing Association
HS(E): Helping Skills Exploration
HS(I): Helping Skills Insight
HS(A): Helping Skills Action
(ESS): Emotional Support Skills
